# Examining the hedge performance of US dollar, VIX, and gold during the coronavirus pandemic: Is US dollar a better hedge asset?

**DOI:** 10.1371/journal.pone.0291684

**Published:** 2023-10-05

**Authors:** Seok-Jun Yun, Sun-Yong Choi, Young Sung Kim

**Affiliations:** 1 Department of Financial Mathematics, Gachon University, Seongnam, Republic of Korea; 2 Head of A.I. Platform Trading Division, SK Securities, Seoul, Republic of Korea; University of Almeria, SPAIN

## Abstract

This study utilizes the hedging potential of the U.S. Dollar Index (USDX) during the COVID-19 period, specifically comparing its positive effects on optimal portfolio weights and hedging ratios with those of traditional hedging assets, such as the VIX and gold. The scalar BEKK GARCH model is employed to forecast volatility and calculate hedging indicators. The results show that USDX exhibits strong hedging abilities against S&P 500 index volatility. These findings highlight the advantageous role of the USDX as a hedging instrument, particularly during periods of heightened market uncertainty, such as during the COVID-19 crisis. Despite the increased market volatility during the COVID-19 pandemic, the value of the optimal portfolio weights is stable and the volatility of the weights is significantly reduced, demonstrating the strength of the USDX’s low risk and volatility in hedging against market fluctuations. Moreover, the increase in the hedge ratio indicates that more capital is allocated to hedging, reflecting the increased correlation between the USDX and S&P 500 index. These results emphasize the beneficial role of the USDX as a hedging instrument during times of elevated market uncertainty, such as during the COVID-19 crisis. Ultimately, USDX can provide valuable insights for market participants seeking effective hedging strategies.

## Introduction

The outbreak of coronavirus (COVID-19, Coronavirus disease 2019, https://www.who.int/emergencies/diseases/novel-coronavirus-2019/technical-guidance/naming-the-coronavirus-disease-(covid-2019)-and-the-virus-that-causes-it) has affected all parts of the world, including the financial market and the real economy ([1]). The pandemic has triggered a recession in many countries. Its impact has been compared with past crises, such as the 2008 global financial crisis (GFC) [2, 3]). According to previous studies, the COVID-19 pandemic shocked financial markets worldwide and was comparable to the 2008 GFC ([4]). Limited economic activity was inevitable, forcing people to enter new economies ([5]). Consequently, the S&P 500 index dropped by almost one-third from the beginning of 2020 ([6]). Before COVID-19, the federal funds rate was 1.75% in January 2020, but the Fed lowered the rate to 0.25% in March 2020. Keeping the rate low for global liquidity but raising the expectation of inflation ([7]). Owing to the backlash caused by COVID-19, the financial market was unpredictable, and conventional hedging could not produce accurate results ([8]). Especially, the pressure of the COVID-19 period on currency volatility increased, making it difficult for the market to react ([9]). For example, KRWUSD reached its highest level of 1280(KRW/USD, the closing price) on March 19, 2020. This was the highest level recorded since the 2008 financial crisis. Since the first exposure to COVID-19, KRWUSD volatility has peaked at 23% (Archived: KRWUSD variance by GARCH model, https://vlab.stern.nyu.edu/volatility). Consequently, equity and currency variances doubled the investor risk ([10]). In addition, rapid movement of currencies has been observed recently (Archived: Globalization Made Rapid Movement of Currencies (https://www.wsj.com/articles/how-investors-can-protect-against-foreign-exchange-exposure-1491790384).

Therefore, this study examined the hedging performance of the US dollar index (USDX), The Chicago Board Options Exchange Volatility Index (VIX), and gold in the US stock market. In particular, we discussed the use of the USDX as a hedging tool for the S&P 500 index. That is, we focused on the hedging performance of the USDX rather than the traditional hedging assets VIX and gold ([11]). Normally, the USDX shows the relative value of the US dollar ([12]). If USDX is rising, it implies the dollar’s value is rising, and vice versa. In finance, the USDX refers to the USD demand. Additionally, we can estimate the relative value of the commodity in US dollars ([13, 14]). The USDX is briefly explained in the US dollar index section.

Several studies have been conducted on portfolio hedging using the USDX. For example, USDX lowers risk returns and diversifies portfolios ([12, 15]). Including USDX in a portfolio can improve returns and reduce risks. Additionally, according to [16], hedging foreign currency risk is often necessary for domestic investors who are exposed to currency volatility. [17] found the safe haven for the emerging stock market by using two hedging tools: Gold and US dollar. Gold provided a protected risk in the usual decline, but US dollar was better at defending against the unusual decline. According to [18], the Russia-Ukraine War has negatively impacted global currency. They have also identified specific currencies that have demonstrated resilience to external shocks. Remarkably, the Canadian dollar has been recognized as a stable currency, supported by its inclusion in the USDX.

Gold and VIX have been extensively studied as hedging assets [19–22]. Numerous studies have investigated their hedging performances in the stock market, including during the COVID-19 pandemic and other crises. For instance, [23] have found that gold was an effective hedge against emerging market risks during normal periods and the GFC. However, as financial markets become more integrated, gold has begun to exhibit movements similar to those of stocks, diminishing its effectiveness as a hedge. Both gold and the VIX are appealing for hedging and diversifying portfolios. However, during the COVID-19 crisis, investors showed a preference for gold over the VIX ([8]). Several studies have examined hedging strategies during the COVID-19 pandemic. [24] have demonstrated that the premium for hedging increased with higher volatility, resulting in investors paying more to achieve the same hedging effect using gold. Moreover, [24] have highlighted that the inclination to hedge increased and impacted the put options of the S&P 500 index and VIX, which reduced the effectiveness of hedging ([25, 26]). Analyzing the hedging effectiveness of gold during the COVID-19 pandemic, [27] have found that a structural collapse, such as COVID-19, has a significant impact on the optimal portfolio weight and hedge ratio. Overall, gold and the VIX have been widely studied as hedging assets, and their effectiveness varies under different market conditions and crises.

Methodologically, we employed the scalar BEKK GARCH model introduced by [28] to describe the relationship between hedge assets and the S&P 500 index. The scalar BEKK GARCH model is an extension of the BEKK GARCH model developed by [29]. The scalar BEKK GARCH model offers several advantages over the original BEKK GARCH model. First, the scalar BEKK GARCH model simplifies the estimation process, making it computationally more efficient. Second, the scalar BEKK GARCH model provides a more parsimonious representation of the conditional covariance dynamics. Accordingly, the scalar BEKK GARCH model has been widely used in financial literature ([30–36]). Based on the estimation results of the scalar BEKK GARCH model, we analyzed their hedging performances (USDX, gold, VIX) in terms of the optimal portfolio weights and hedge ratios. The sample period was set from January 2020 to June 2022, including the COVID-19 pandemic.

This study examined equity holders’ hedging performance during the COVID-19 pandemic using the US dollar index, VIX, and gold. VIX and gold are considered classic hedging tools. This study differs from other studies in its use of the USDX as a hedging tool. Especially during the COVID-19 pandemic, the demand for US dollars has increased. The purpose of this study is to investigate whether the USDX can be a safe haven alternative to equities and its role in times of market turmoil, such as the COVID-19 pandemic. There are some studies on safe haven assets other than equities in the USDX ([37–39]), but to the best of our knowledge, no studies have been conducted on the S&P 500 Index.

The remainder of this paper is organized as follows. In the following section, we introduce existing literature related to our research. The Data Description section describes the data and provides a preliminary statistical analysis of the S&P 500 index, USDX, gold and VIX. In the Methodology section, we review the scalar BEKK GARCH method, optimal portfolio weights, and hedge ratios. The Empirical Results section presents the empirical results and the data analysis. The Discussion and Concluding Remarks section presents the summary and concluding remarks.

## Literature review

### COVID-19 pandemic and financial markets

Several studies have examined the impact of the COVID-19 pandemic on financial markets. [40] have found that the pandemic reduced risk exposure to oil prices because of decreased demand. Additionally, [41] have found that the crisis increased financial contagion, with stock prices becoming more correlated across countries. [42] have found that COVID-19 had significant and short-term impacts on financial markets worldwide, with a greater impact on Asian markets than on developed markets. Finally, [43] have demonstrated that ESG leader indices outperformed non-ESG leader indices during the pandemic, indicating investor confidence in the resilience of ESG-oriented companies. [44] have shown that airline stocks fell because of COVID-19 and were expected to rise with the advent of a cure, but it took time. With low-cost carriers exhibiting a more resilient rebound. [45] analyzed the return, volatility, and bad state probability dynamics during COVID-19 and found that the negative impact of deaths was greater than the recovery from COVID-19 across the US, Europe, and Asia. [46] have examined spillover effects from COVID-19 in 51 countries and found that continents with high GDP, such as the Eurozone, Asia, and North America, stood out. They have also conducted a comparative analysis of emerging market stocks during the GFC and COVID-19. [47] have found that the volatility of the currency exchange rate affects the stock market, which is why the central bank’s stabilization of the currency exchange rate plays an important role in investor confidence.

### COVID-19 pandemic and safe haven

Many studies have explored the role of various safe haven assets. Silver and gold have been found to be effective hedges against policy uncertainty and COVID-19 in the Chinese market, with gold exhibiting greater effectiveness. Additionally, silver is a valuable hedge against inflation ([48]). Cryptocurrencies are identified as effective hedges against EPU (Economic Policy Uncertainty) and equity market volatility, particularly in times of heightened uncertainty ([49]). [50] have investigated the potential of gold, oil, equities, and currencies as hedges against EPU and GPR (Geopolitical Risks) during the COVID-19 crisis, although their effectiveness was limited under extreme uncertainty and risk. [51] have studied the role of gold in European equity and bond markets during COVID-19. They have found that gold was not as effective during the GFC. [52] have analyzed green bonds as a safe haven during COVID-19. They have found that green bonds could be a diversifying asset for investors and have the potential to be a safe haven during times of market volatility. [53] have also found that green bonds could be a safe haven, but noted that they were not as effective as hedging financial sectors. [54] have examined insurance companies in India and Taiwan to see if they were safe havens during COVID-19. They have found that insurance futures served as a safe haven for the markets in these two countries, which suffered from high rates of infection.

### Conflicts and stock markets

Numerous studies have examined the effects of disputes, including military actions, on the stock market ([55–61]). For instance, [60] have investigated the impact of terrorism on local stock markets in G7 countries. Their findings have revealed that stock markets experience significant losses in the aftermath of terrorist events. Similarly, [61] have demonstrated that border disputes can have substantial adverse effects on economic growth, investment, and trade.

Recently, the Russia-Ukraine War (RU-war) has had a significant negative impact on global stock markets. According to [62], this effect is particularly pronounced in countries with stronger economic connections with Russia and Ukraine. [63] have further explained that the effects of the RU-war on global equity markets have been heterogeneous, with developed markets experiencing a higher level of impact. [64] have examined the impact of the RU-war on Russia, European stock markets, and global commodity markets. They have found that the war has altered the interconnectedness among these markets. Specifically, according to [65], the European Union stock markets are particularly vulnerable to the consequences of the war, and prolonged conflict could significantly harm the region’s economy. Furthermore, [66] have used an event study approach to assess the impact of the RU war on G20 stock markets. Their findings have indicated that the war negatively influenced stock markets, both on the event day (February 24, 2022) and on the days following the event. As a result of the RU-war, investors have become more concerned with security and stability, leading to a decline in investments in risky assets ([67]).

## Data description

We set the period for our analysis from January 1, 2018, to June 30, 2022, representing the entire duration of the study. To identify a more effective hedging tool for the COVID-19 period, we divide the study into the following two distinct periods: Periods 1 and 2. The base date for this division is set at the beginning of 2020(WHO Timeline, COVID-19, https://www.who.int/news/item/27-04-2020-who-timeline---covid-19).

By the first quarter of 2020, COVID-19 had spread globally and financial asset markets had crashed significantly ([68]). For the equity market, we used the S&P 500 for daily closing data from New York Stock Exchange (NYSE). To compare the performance of hedge assets, we used VIX from the Chicago Board Options Exchange (CBOE) and gold from the Commodity Exchange Inc. (COMEX) for the same period of daily closing data. In addition, we used the US dollar index quoted on the Intercontinental Exchange (ICE). The US Dollar Index was started at a value of 100 in 1973. All data were obtained from Yahoo Finance.

The second period refers to the strength of the COVID-19 pandemic period (January 1, 2020 to June 30, 2022). In the second period, the minimum value of the S&P 500 is -0.1277; however, the first period (January 1, 2018 to December 31, 2019) has a minimum value of -0.0418. This demonstrates how hard the COVID-19 period was to react. Moreover, the standard deviation increased by 74.47%, from 0.0094 to 0.0164.

The USDX has also been affected by COVID-19. In period 2, under the influence of COVID-19, the value for Jarque–Bera was 52.13. Jarque–Bera had its lowest value of 0.3426 in period 1, which was not under the influence of COVID-19. This resembles a normal distribution. The group of assets with less volatility or movement, such as the USDX, also changed. This is a definite indication of the impact of COVID-19 on financial markets.

The VIX also indicates the period of turmoil. The mean VIX increased from 0.007 to 0.0012 during the COVID-19 period. Interestingly, the Jarque-Bera test statistic decreased from 3302 to 638, indicating that the VIX exhibited a distribution closer to normal during this time. Among the four asset classes, gold stands out as the only one with a change in the skewness sign caused by COVID-19. In the unaffected period, it has a skewness of 0.2030, whereas in the COVID-19-affected period, it exhibits a skewness of -0.3643.

**Fig 1:**
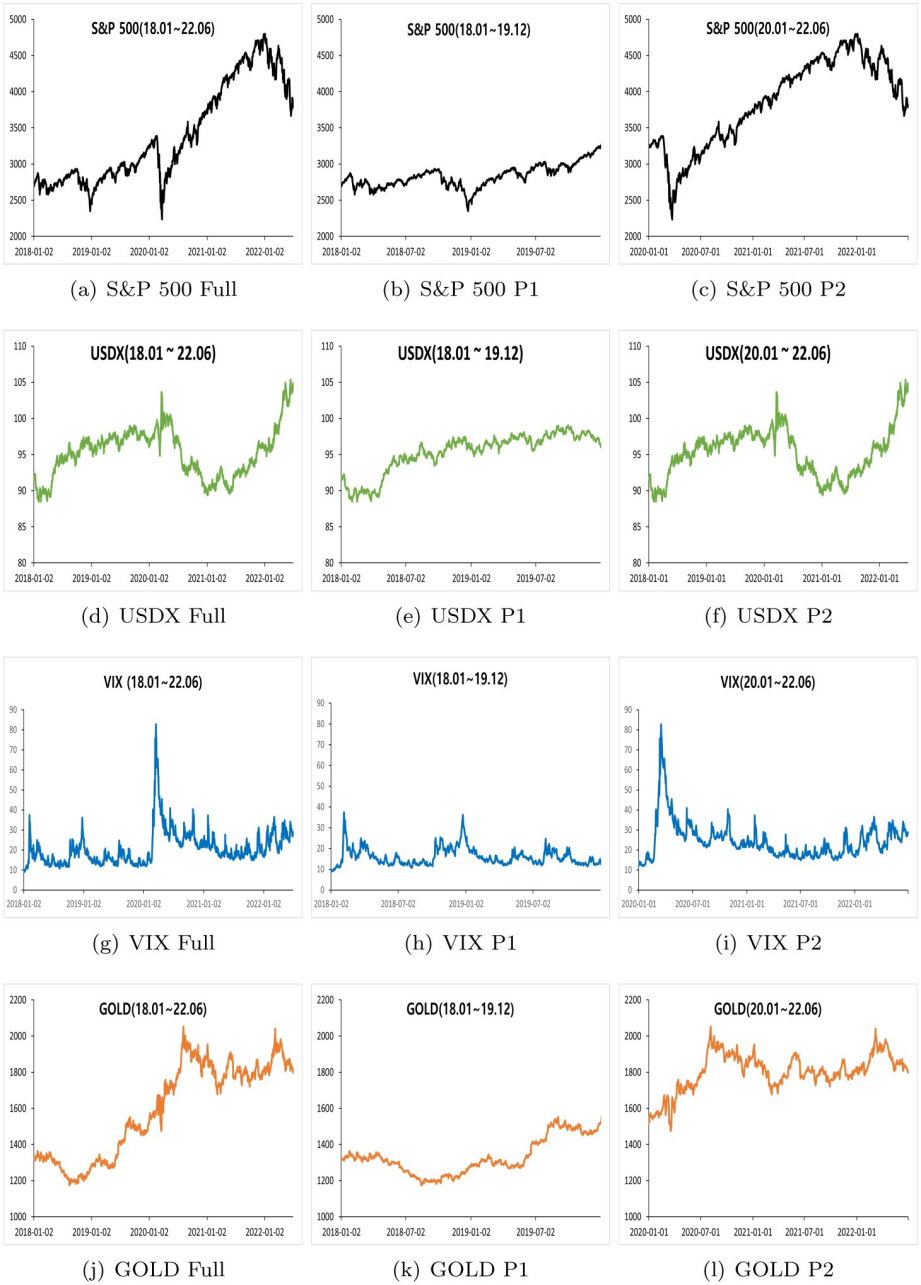
Full period, Period 1 and Period 2. ‘Full’ means the time period from January 1, 2018, to June 30, 2022.

Fig 1 illustrates the dynamics of the four asset prices. The full period indicates the cumulative sum of period 1 (January 1, 2018 to December 31, 2019) and period 2 (January 1, 2020 to June 30, 2022). Fig 1(a) presents an overall graph of the S&P 500. Owing to COVID-19, a large drop was observed in early 2020. The intensity of the decline was rapid and the angle was sharp; however, it took some time to recover and reach a new height. After it was reached, the downward pressure was applied to the inflationary pressure (“Hike of FED rate and pressure to S&P 500”, https://www.wsj.com/articles/global-stocks-markets). Fig 1(d) is an overall graph of the USDX. A novel trend was detected during the first quarter of 2020. Prices did not significantly change before the influence of COVID-19 prevailed in the market. Fig 1(g) shows a general graph of VIX. The VIX shows a significant increase when there is a large decrease in the S&P 500. In particular, the S&P 500 showed a series of declines through COVID-19, VIX hit a record high of 82.69pt on March 16, 2020 ([21, 69, 70]). Fig 1(j) shows the overall graph of gold. Gold rose at the beginning of the COVID-19 period, but it was not noticeable to hedge by moving significantly in the opposite direction to the S&P 500 ([71, 72]).

Specifically, at the beginning of the significant decline in the S&P 500, the USDX fell along with it and reached 94.87pt on March 9, 2020. However, the situation in the dollar index was reversed. It reached 103.50pt on March 20, 2020. The USDX fluctuated by an average of 0.01% per day (over the entire period). In 10 business days, it moved by 9.10%. That is 0.91% per day. As the price of the S&P 500 rebounds, the USDX slowly fell below 90 and began to rise in June 2021. Daily change(%) data show that the correlation between the entire period of the S&P 500 and the dollar index is -0.1051. However, when we calculated the correlation for the COVID-19 period (January 1, 2020 to June 30, 2022), it almost doubled to -0.2360.

## Methods

### Scalar BEKK-GARCH model

In this section, we briefly introduce the scalar BEKK GARCH model proposed by [28]. Additionally, we assume that the return process follows the VAR (Vector Auto Regression) model. Accordingly, the conditional mean equation(VAR(*p*)) is defined as

$$\begin{array}{c} r_{t}=\mu +\sum_{i=1}^{p}\Gamma_{i}r_{t-i}+\epsilon_{t} \end{array}$$ (1)

where *r*_*t*_ is an *n* × 1 return vector for *n* different assets, *μ* is the *n* × 1 constant vector, *γ*_*i*_ are *n* × *n* coefficient matrices. *ϵ*_*t*_ is an *n* × 1 vector of zero-mean error terms with conditional covariance matrix *H*_*t*_, i.e. $\epsilon_{t}=\sqrt{H_{t}}\nu_{t}$. *ν*_*t*_ is an *n* × 1 vector of standardized residuals.

For the conditional variance–covariance equations, the conditional covariance matrix of the scalar BEKK model, *H*_*t*_, is expressed as

$$\begin{array}{c} H_{t}=C^{\prime }C+\alpha \epsilon_{t-1}\epsilon_{t-1}^{\prime }+\beta H_{t-1}, \end{array}$$ (2)

where *H*_*t*_ is the *n* × *n* covariance matrix; *C* is a (*n* × *n*) upper triangular matrix. The constant *α* and *β* are ARCH and GARCH parameters, respectively. Furthermore, *ϵ*_*t*−1_ is the *n* × 1-vector of the error terms in (1).

In this study, we used a two-variate(*n* = 2) scalar BEKK GARCH(1,1) model written as

$$\begin{array}{ccc} & & (\begin{array}{cc} h_{11,t} & h_{12,t}\\ h_{21,t} & h_{22,t} \end{array})=\\ & & (\begin{array}{cc} c_{11} & c_{12}\\ 0 & c_{22} \end{array}{)}^{\prime }(\begin{array}{cc} c_{11} & c_{12}\\ 0 & c_{22} \end{array})+\alpha (\begin{array}{cc} \epsilon_{1,t-1}^{2} & \epsilon_{1,t-1}\epsilon_{2,t-1}\\ \epsilon_{2,t-1}\epsilon_{1,t-1} & \epsilon_{2,t-1}^{2} \end{array})+\beta (\begin{array}{cc} h_{11,t-1} & h_{12,t-1}\\ h_{21,t-1} & h_{22,t-1} \end{array}). \end{array}$$ (3)

The matrix equation of the scalar BEKK GARCH(1,1) model (3) is derived from the BEKK GARCH model. Further details of the scalar BEKK estimation process have been presented in previous studies ([33, 35]).

### Optimal portfolio weights and hedge ratios

Suppose that an investor holds asset *S* and wants to hedge his exposure to unfavorable movements in asset *H*. Following [29], optimal portfolio weights can be constructed.

$$\begin{array}{c} W_{SH,t}=\frac{h_{H,t}-h_{SH,t}}{h_{S,t}-2h_{SH,t}+h_{H,t}} \end{array}$$ (4)

$$\begin{array}{c} W_{SH,t}=\{\begin{array}{cc} 0 & \text{if}\;W_{SH}{,}_{t}<0\\ W_{SH,t} & \text{if}\;0\leq W_{SH,t}\leq 1\\ 1 & \text{if}\;W_{SH,t}>1 \end{array} \end{array}$$ (5)

For the optimal weight for the same amount portfolio of stock(S&P 500) / hedging assets (i.e., USDX, VIX, gold in this study) at time *t*, where *h*_*H*,*t*_ denotes the conditional variance of the hedging tool. *h*_*S*,*t*_ is the conditional variance of the stock in this study, S&P 500. *h*_*SH*,*t*_ is the conditional covariance between stock and hedging assets.

$$\begin{array}{c} \beta_{SH,t}=\frac{h_{SH,t}}{h_{H,t}} \end{array}$$ (6)

The hedge ratio defines the extent to which a one-dollar-long position in the stock market (S&P 500) must be hedged by a short position in the hedge asset market (i.e., USDX, VIX, gold).

If *β*_*SH*,*t*_ is positive, the long position in the risk asset is hedged by the short position in the hedge asset. A negative *β*_*SH*,*t*_ indicates that optimal hedging implies a long position for both equity and hedging assets.

## Empirical results

We conduct stationarity tests, including the Augmented Dickey-Fuller (ADF), Phillips-Perron (PP), and Kwiatkowski-Phillips-Schmidt-Shin (KPSS) tests on the USDX, gold, VIX, and S&P 500 assets. These tests are performed to determine the presence of a unit root in the asset returns, which is essential for estimating the optimal portfolio weights and hedge ratios.

Table 1 shows the results of the ADF, PP, and KPSS unit-root tests for the log–return series. The test results indicate that USDX, gold, VIX, and S&P 500 assets are stationary, implying that they tend to exhibit trends over time.

**Table 1:** The results of the ADF, PP, and KPSS unit root tests.

Panel C: Results of the Unit Root Tests			
Asset	ADF	PP	KPSS
S&P 500	-41.177***	-40.663***	0.0878*
USDX	-32.636***	-32.626***	0.1882*
Gold	-34.021***	-34.201***	0.1051*
VIX	-37.030***	-37.296***	0.0257*

Notes: The table presents the results for the ADF, PP, and KPSS unit root tests for the four return series from January 1, 2018, to June 30, 2022. ADF, PP, and KPSS are Augmented Dickey-Fuller, Phillips-Perron, and Kwiatkowski-Phillips-Schmidt-Shin, respectively.

(***) and (*) indicates a rejection of the null hypothesis at the 1% and 10% significance level, respectively.

When estimating the VAR model, it is crucial to determine the appropriate lag order using various model selection criteria ([73]). These criteria help estimate different VAR(*p*) models, and the model with the lowest information criterion is considered the most suitable. By comparing different information criteria–the Akaike Information Criterion (AIC), HQ–Quinn information criterion (HQ), Schwarz Bayesian Information Criterion (SC), and Final Prediction Error Criterion (FPE)–we can identify the VAR model that provides the best fit for the data. Table 2 lists the VAR model’s estimation results. The AIC, SC, HQ, and FPE information criteria are used to determine the lag order of the VAR model. For the lag 10 order, the information criterion values of each lag period are shown in Table 2. Accordingly, we select an optimal lag of 1. The estimation results of VAR are given in Table 3.

**Table 2:** VAR lag order selection criteria.

Order	AIC	HQ	SC	FPE
1	-34.9141*	-34.8802*	-34.8245*	6.87E-16
2	-34.9680	-34.9341	-34.8783	6.51E-16
3	-34.9952	-34.9613	-34.9054	6.34E-16
4	-35.0452	-35.0113	-34.9554	6.03E-16
5	-35.0874	-35.0535	-34.9975	5.78E-16
6	-35.1293	-35.0953	-35.0393	5.54E-16
7	-35.1981	-35.1640	-35.1080	5.17E-16
8	-35.2357	-35.2017	-35.1456	4.98E-16
9	-35.2768	-35.2427	-35.1866	4.78E-16
10	-35.2908	-35.2567	-35.2005	4.71E-16*

Notes: The table gives the lag length selection criteria.

(*) denotes the optimal lag order selected by the information criterion. Notes: AIC, HQ, SC and FPE are Akaike Information Criterion, Hannan-Quinn Information Criterion, Schwarz Bayesian Information Criterion and Final Prediction Error Criterion, respectively.

**Table 3:** The conditional mean equation and scalar BEKK-GARCH model parameter estimates for the three portfolios.

S&P 500 / USDX Portfolio				
Conditional mean equation		Scalar BEKK GARCH		
*μ* (2 × 1)	Γ (2 × 2)	C (2 × 2)	*α*	*β*
$\left(\begin{array}{c} \underset{\left(0.987\right)}{0.0003}\\ \underset{\left(1.222\right)}{0.0001} \end{array}\right)$	$\left(\begin{array}{cc} \underset{\left(-7.157\right)}{-0.2091^{‡}} & \underset{\left(-2.841\right)}{-0.2996^{‡}}\\ \underset{\left(-8.854\right)}{-0.0709^{‡}} & \underset{\left(0.133\right)}{0.0038} \end{array}\right)$	$\left(\begin{array}{cc} \underset{\left(4.7920\right)}{0.0018^{‡}} & \underset{\left(-0.9144\right)}{-7.04E-05}\\ 0 & \underset{\left(5.4317\right)}{0.0007^{‡}} \end{array}\right)$	$\underset{5.1054}{0.0930^{‡}}$	$\underset{33.4489}{0.8771^{‡}}$
S&P 500 / Gold Portfolio				
Conditional mean equation		Scalar BEKK GARCH		
*μ* (2 × 1)	Γ (2 × 2)	C (2 × 2)	*α*	*β*
$\left(\begin{array}{c} \underset{\left(0.831\right)}{0.0003}\\ \underset{\left(0.981\right)}{0.0002} \end{array}\right)$	$\left(\begin{array}{cc} \underset{\left(-6.988\right)}{-0.2037^{‡}} & \underset{\left(2.057\right)}{0.0854^{†}}\\ \underset{\left(0.099\right)}{0.0021} & \underset{\left(-0.441\right)}{-0.0131} \end{array}\right)$	$\left(\begin{array}{cc} \underset{\left(5.7621\right)}{0.0019^{‡}} & \underset{\left(-0.3718\right)}{-6.39E-05}\\ 0 & \underset{\left(6.4332\right)}{0.0021^{‡}} \end{array}\right)$	$\underset{5.5469}{0.1076^{‡}}$	$\underset{36.0558}{0.8584^{‡}}$
S&P 500 / VIX Portfolio				
Conditional mean equation		Scalar BEKK GARCH		
*μ* (2 × 1)	Γ (2 × 2)	C (2 × 2)	*α*	*β*
$\left(\begin{array}{c} \underset{\left(1.031\right)}{0.0004}\\ \underset{\left(0.285\right)}{0.0007} \end{array}\right)$	$\left(\begin{array}{cc} \underset{\left(-7.334\right)}{-0.3092^{‡}} & \underset{\left(-3.534\right)}{-0.0232^{‡}}\\ \underset{\left(3.195\right)}{0.8762^{‡}} & \underset{\left(0.053\right)}{0.0022} \end{array}\right)$	$\left(\begin{array}{cc} \underset{\left(7.6128\right)}{0.0025^{‡}} & \underset{\left(-7.0261\right)}{-0.0207^{‡}}\\ 0 & \underset{\left(8.7826\right)}{0.0151^{‡}} \end{array}\right)$	$\underset{5.7936}{0.1702^{‡}}$	$\underset{25.1433}{0.7773^{‡}}$

Notes: The table displays the estimation results of conditional mean and variance for the three portfolios. The *t*-values of the estimated parameters are displayed in parentheses. ^†^ and ^‡^ indicate a rejection of the null hypothesis at the 5% and 1% significance level, respectively.

This section focuses on the construction of three portfolios, namely, S&P 500 / USDX, S&P 500 / Gold, and S&P 500 / VIX, and the determination of their optimal portfolio weights and hedge ratios using the scalar BEKK GARCH model. The estimation results of the model are presented in Table 3. Additionally, Table 4 presents various evaluation metrics, including the log-likelihood values, information selection criteria, and diagnostic test results based on standard residuals (*ν*_*t*_).

**Table 4:** The Goodness-of-fit results and standardized residual diagnostics for the three portfolios.

Portfolio	S&P 500 / USDX		S&P 500 / Gold		S&P 500 / VIX	
AIC	-16649.69		-14528.38		-10830.25	
SC	-16633.66		-14512.29		-10814.16	
Log Likelihood	8328.344		7267.69		5418.625	
Standardized residual diagnostics						
Portfolio	S&P 500 / USDX		S&P 500 / Gold		S&P 500 / VIX	
*ν*_*t*_	S&P 500	USDX	S&P 500	Gold	S&P 500	VIX
Mean	0.0019	0.0064	0.003	-0.0045	0.0066	0.0034
Std.Dev.	1.0077	0.9924	1.0095	0.9877	1.0131	0.9862
Skewness	-0.9854	-0.1321	-0.9666	-0.5378	-0.974	0.6263
Kurtosis	2.42	1.1815	2.2676	3.5007	2.3247	4.0844
*Q*(40)	52.2167	42.0068	54.4136	36.4601	34.0578	63.8538^‡^
*Q*^2^(40)	49.1942	39.9862	44.6713	40.5795	36.0291	38.3461
ARCH(40)	44.668	52.729	42.455	38.86	34.622	38.054

Notes: *Q*(40) and *Q*^2^(40) indicate the Ljung-Box Q-statistic of order 40 computed on the standardized residuals and squared standardized residuals, respectively. ARCH(40) is the non-heteroskedasticity statistic of order 40 of the ARCH Engle test.

^†^ and ^‡^ indicate a rejection of the null hypothesis at the 5% and 1% significance level, respectively.

As depicted in Table 4, the analysis reveals that we do not have sufficient evidence to reject the null hypothesis of no autocorrelation for the standardized and squared standardized residuals. This outcome implies that our chosen scalar BEKK GARCH parameterization adequately captures the conditional variance for each portfolio.

**Fig 2:**
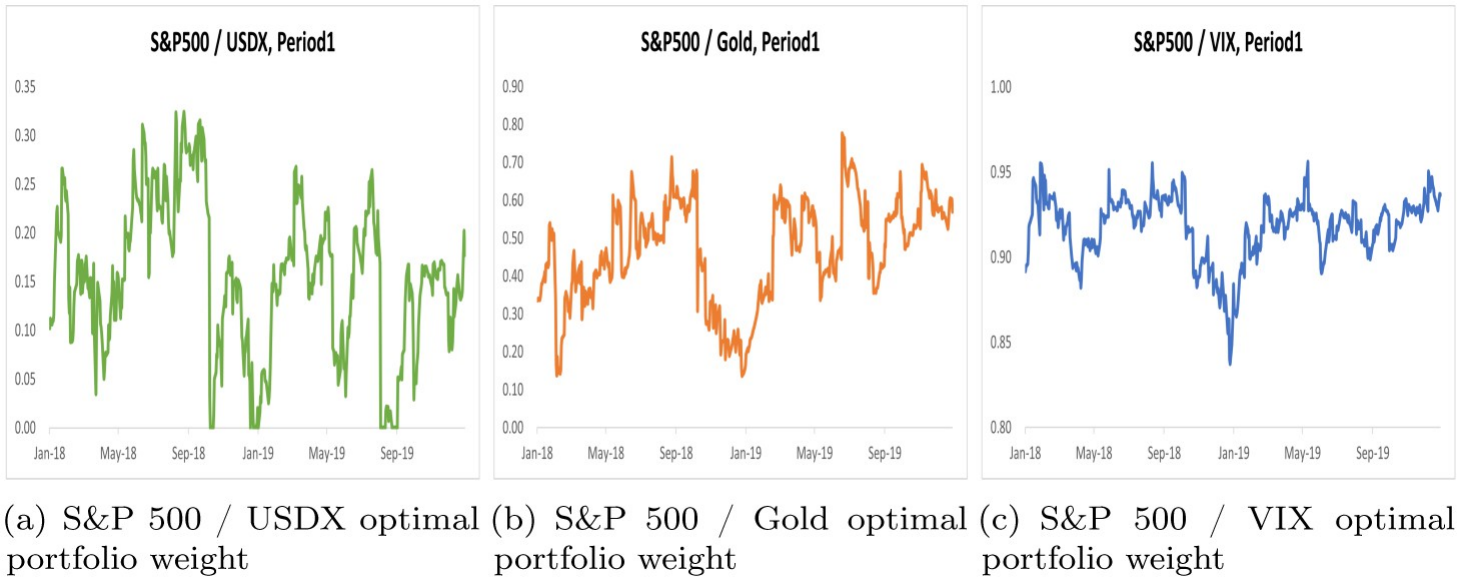
Optimal portfolio weight on Period 1.

**Fig 3:**
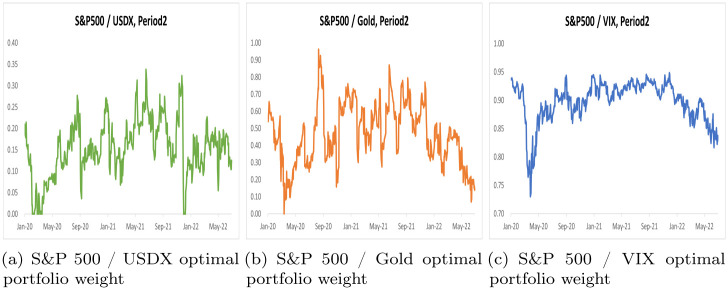
Optimal portfolio weight on Period 2.

**Fig 4:**
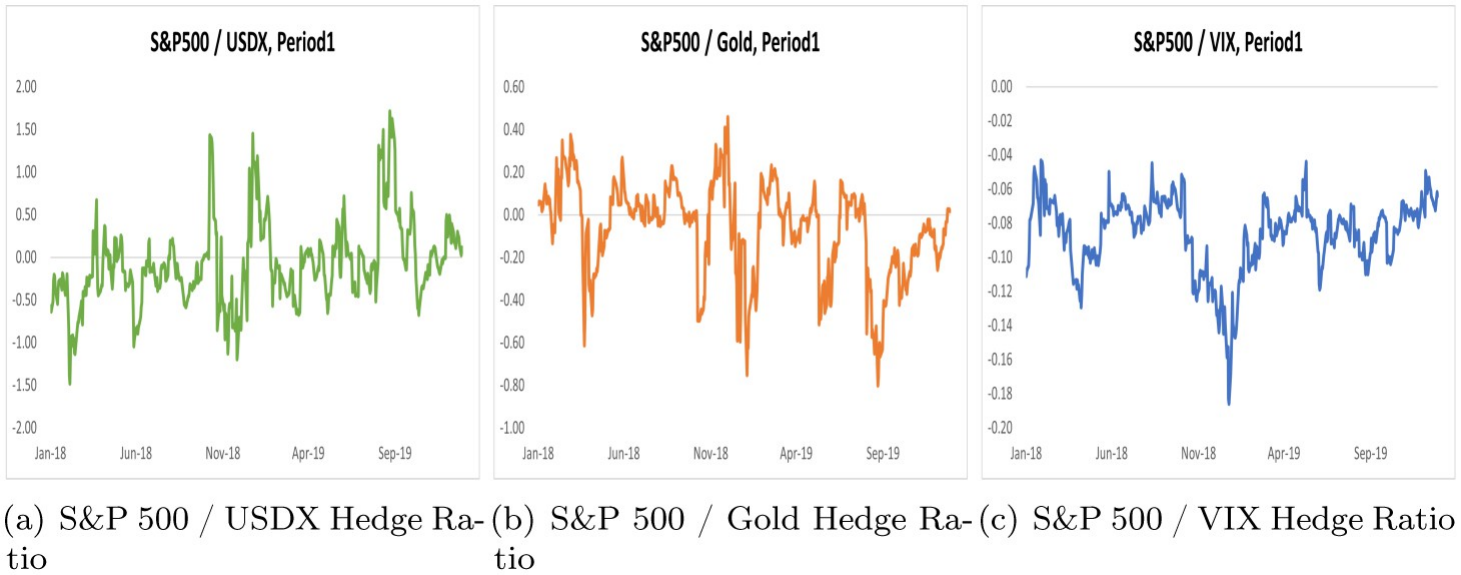
Hedge ratio on Period 1.

**Fig 5:**
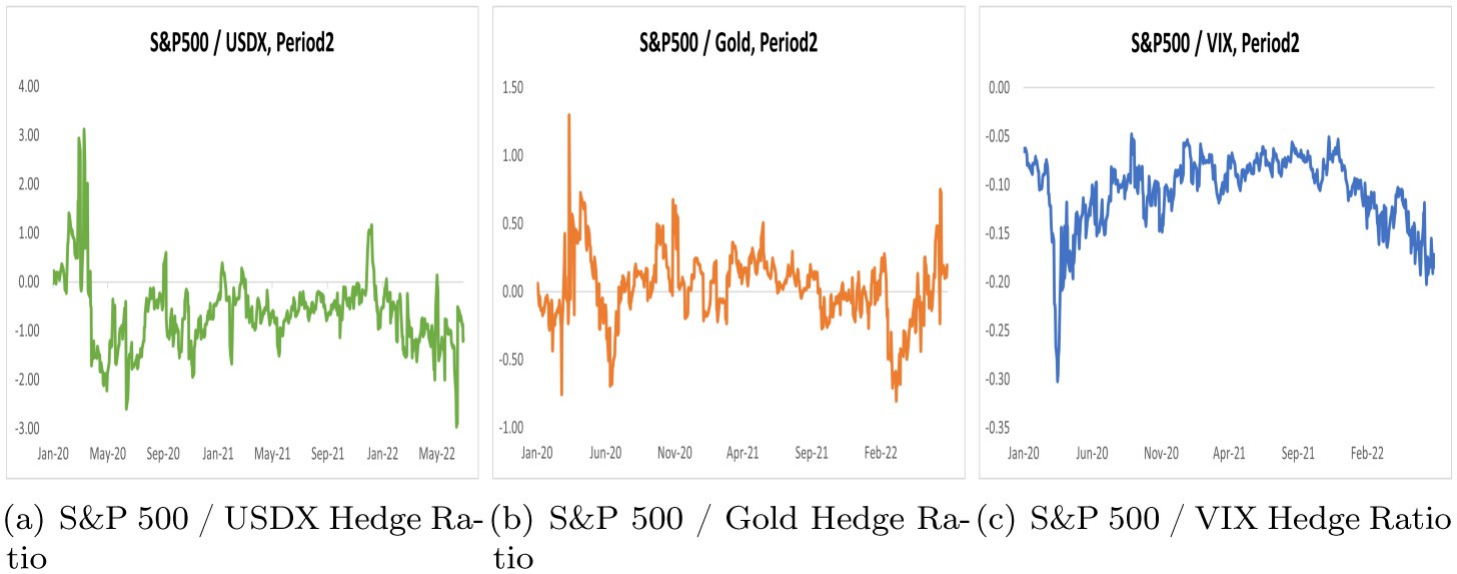
Hedge ratio on Period 2.

By using the scalar BEKK GARCH model, we computed optimal portfolio weights and hedge ratios for two distinct periods, Period 1 and Period 2, across three portfolios. These results are summarized in Tables 5 and 6, while Figs 2 and 3 illustrate optimal portfolio weights for Period 1 and Period 2. Additionally, Figs 4 and 5 depict hedge ratios for the corresponding periods.

**Table 5:** Summary statistics of optimal portfolio weights.

Panel D: Summary statistics of optimal portfolio weights on Period 1.				
Portfolio	Mean	Std.Dev	Min.	Max.
S&P 500 / USDX	0.1541	0.0777	0	0.3242
S&P 500 / Gold	0.4675	0.1345	0.1370	0.7761
S&P 500 / VIX	0.9181	0.0186	0.8374	0.9561
Panel E: Summary statistics of optimal portfolio weights on Period 2.				
Portfolio	Mean	Std.Dev	Min.	Max.
S&P 500 / USDX	0.1531	0.0657	0	0.3380
S&P 500 / Gold	0.4670	0.1755	0	0.9620
S&P 500 / VIX	0.8976	0.0361	0.7305	0.9485

Notes: The table shows the summary statistics of optimal portfolio weights for the three portfolios during the two periods.

**Table 6:** Summary statistics of hedge ratio.

Panel F: Summary statistics of Hedge Ratio on Period 1.				
Portfolio	Mean	Std.Dev	Min.	Max.
S&P 500 / USDX	-0.0833	0.4953	-1.4764	1.7123
S&P 500 / Gold	-0.0729	0.2211	-0.8005	0.4583
S&P 500 / VIX	-0.0859	0.0216	-0.1858	-0.0434
Panel G: Summary statistics of Hedge Ratio on Period 2.				
Portfolio	Mean	Std.Dev	Min.	Max.
S&P 500 / USDX	-0.6335	0.7455	-2.9587	3.1177
S&P 500 / Gold	0.0309	0.2563	-0.8011	1.2956
S&P 500 / VIX	-0.1065	0.0375	-0.3017	-0.0482

Notes: The table shows the summary statistics of optimal portfolio weights for the three portfolios during the two periods.

Based on the estimation results, the empirical findings are as follows. First, the value of the optimal portfolio weight allows us to calculate the amount of the S&P 500 index that we should own to create an optimal portfolio. According to Table 5, the optimal portfolio weight for the S&P 500 / USDX portfolio is 0.1541, which means that one should buy 0.1541 for the S&P 500 and 0.8459 for USDX (= 1-0.1541) to create an optimal portfolio.

What makes USDX a good choice for an optimal portfolio is that its value does not fluctuate significantly. Gold and VIX have higher volatility than USDX. As shown in Table 5, USDX has a standard deviation difference of negative 15.44%, from 0.0777 to 0.0657 from period 1, which is the normal time frame, to period 2, which was affected by COVID-19. However, the VIX records a standard deviation change of 194.09% and gold records a standard deviation change of 30.48%. Despite being affected by COVID-19, USDX in the optimal portfolio weights shows that it is an asset to watch closely.

By contrast, the optimal portfolio weight values for the S&P 500 / VIX portfolio are 0.9181 and 0.8976 in periods 1 and 2, respectively. This means that buying VIX does not have a significant impact on the optimal portfolio in both periods. The reason why the value of the optimal portfolio weight is close to 1 can be explained by the fact that VIX is inversely correlated with the S&P 500 ([74]).

For the S&P 500 / Gold portfolio, the optimal portfolio weight is 0.4675 in period 1 and 0.4670 in period 2, showing that having more than 50% gold in the portfolio for all periods helps create an optimal portfolio. Additionally, as the value decreases in period 2, gold is a safe haven for building an optimal portfolio during COVID-19 ([8, 75, 76]).

If the optimal portfolio weight is based solely on buying hedges, then the hedge ratio is split between selling and buying assets. In other words, a negative number indicates how selling an asset can affect the portfolio.

Based on the hedge ratio using the scalar BEKK GARCH model (Table 6), we can see that for a $1 purchase of the S&P 500, the USDX must be short by -0.0833 in period 1 and -0.6335 in period 2. As the value of USDX increases in absolute terms during the COVID-19 period, more capital is used for hedging. However, gold’s hedge ratio indicates that it was able to hedge the S&P 500 with less value during COVID-19, as gold’s hedge ratio was -0.0729 in period 1, but 0.0309 in period 2. The sign of the hedge ratio changed, indicating that gold changed its characteristic during COVID-19. The VIX shows a hedge ratio of -0.0859 in Period 1, but a value of -0.1065 in Period 2 during COVID-19. Considering the hedge ratio, gold has less capital to hedge in the S&P 500 during the COVID-19 pandemic.

Furthermore, using the Kruskal-Wallis test, we examine whether the three portfolios are grouped together and whether the median values of each portfolio are statistically different. Table 7 presents the results of the Kruskal-Wallis test. The table indicates significant differences in the median values among the portfolios for the full period and Periods 1 and 2. This confirms that each portfolio shows significant differences in hedging behavior in terms of median values of optimal portfolio weights and hedge ratios. Moreover, Table 8 shows that the optimal portfolio weights and hedge ratios of the three portfolios have significantly different median values for Periods 1 and 2, except for the optimal portfolio weights of the S&P 500 / Gold portfolio.

**Table 7:** The results of the Kruskal-Wallis test on the optimal portfolio weights and hedge ratio of the three portfolios.

Panel H: Kruskal-Wallis test on full period comparing total 3 portfolios		
Portfolio	Chi-square Statistics	Null hypothesis
Optimal portfolio weights	2830.8	Reject at the 1 percent level
Hedge ratio	1181.2	Reject at the 1 percent level
Panel I: Kruskal-Wallis test on period 1 comparing total 3 portfolios		
Portfolio	Chi-square Statistics	Null hypothesis
Optimal portfolio weights	1276.1	Reject at the 1 percent level
Hedge ratio	590.0	Reject at the 1 percent level
Panel J: Kruskal-Wallis test on period 2 comparing total 3 portfolios		
Portfolio	Chi-square Statistics	Null hypothesis
Optimal portfolio weights	1582.7	Reject at the 1 percent level
Hedge ratio	638.1	Reject at the 1 percent level

Notes: The table displays the outcomes of the Kruskal-Wallis test conducted on the optimal portfolio weights and hedge ratio for the three portfolios across the full period, as well as within Periods 1 and 2. The Chi-square Statistics are the difference between the mean values. The null hypothesis states that the median of the groups is equal.

**Table 8:** The results of the Kruskal-Wallis test on the optimal portfolio weights and hedge ratio of each portfolio.

Panel K: Kruskal-Wallis test on each optimal portfolio weights comparing period 1 and 2		
Portfolio	Chi-square Statistics	Null hypothesis
S&P 500 / USDX	45.615	Reject at the 1 percent level
S&P 500 / Gold	0.023	Accept
S&P 500 / VIX	94.497	Reject at the 1 percent level
Panel L: Kruskal-Wallis test on each hedge ratio comparing period 1 and 2		
Portfolio	Chi-square Statistics	Null hypothesis
S&P 500 / USDX	23.634	Reject at the 1 percent level
S&P 500 / Gold	61.370	Reject at the 1 percent level
S&P 500 / VIX	86.111	Reject at the 1 percent level

Notes: The table presents the Kruskal-Wallis test results for the optimal portfolio weights and hedge ratio values across Periods 1 and 2 of each portfolio.

## Discussion and concluding remarks

In this study, we compared the optimal portfolio weights and hedge ratios for the S&P 500 Index to USDX, VIX, and gold using daily return data for the periods before and during COVID-19 to determine which hedging assets were significantly useful during the COVID-19 period. The scalar BEKK GACRH model is employed to describe portfolio volatility. Furthermore, using the Kruskal–Wallis test, we show that the three portfolios have significantly different optimal portfolio weights and hedge ratios.

First, USDX proved to be a strong part of the optimal portfolio weights because of its ability to reduce the risk of the S&P 500. This is evidenced by a reduction in the optimal portfolio weights of the S&P 500 (Table 5) from 0.1541 to 0.1531 during the COVID-19 period. Compared to traditional hedging assets, such as VIX or gold, it can be seen as a good way to hedge against market volatility. In the hedge ratio (Table 6), the USDX and VIX both worked as hedging assets against volatility; selling the USDX or buying the VIX reduced the volatility of the S&P 500 across both periods. However, gold is a more complicated hedging asset because selling gold helps reduce volatility in Period 1, whereas buying gold helps reduce volatility in Period 2.

Second, in terms of the hedge ratio, the USDX requires a larger amount of capital than gold and the VIX in Period 2. Therefore, to achieve the same level of volatility hedge, more capital would need to be allocated to the USDX than to gold and the VIX. Therefore, when considering the hedge ratio, the USDX is considered an inefficient asset class compared to the VIX and gold. However, in Period 1, the VIX has the largest hedge ratio for the same level of volatility.

Third, in the overall market statistics (Table 9), the standard deviation of the S&P 500’s periods 1 and 2 increases by 74.47% (from 0.0094 to 0.0164) but the standard deviation of USDX’s optimal portfolio weights decreases by 15.44% (from 0.0777 to 0.0657). In addition, the hedge ratio for USDX increased by 50.51% (from 0.4953 to 0.7455). Consequently, USDX is an asset class that can have a positive effect on reducing the volatility of a portfolio compared with other traditional hedge asset classes.

**Table 9:** Summary statistics for the log return of S&P 500 index, VIX, USDX, and gold.

Panel A: Non affected period (January 1, 2018 to December 31, 2019, Period 1(502 days))							
Sectors	Mean	Max.	Min.	Std.Dev.	Skewness	Kurtosis	J-B.
S&P 500 index	0.0004	0.0484	-0.0418	0.0094	-0.6141	3.6931	317^‡^
USDX	0.0001	0.0112	-0.0101	0.0034	0.0157	0.1241	0.3426^‡^
Gold	0.0003	0.0350	-0.0230	0.0070	0.2030	2.4286	127^‡^
VIX	0.0007	0.7682	-0.2596	0.0884	1.8533	12.0043	3302^‡^
Panel B: COVID-19 affected period(January 1, 2020 to June 30, 2022, Period 2(629 days))							
Sectors	Mean	Max.	Min.	Std.Dev.	Skewness	Kurtosis	J-B.
S&P 500 index	0.0003	0.0897	-0.1277	0.0164	-0.8945	12.1238	3936^‡^
USDX	0.0001	0.0159	-0.0170	0.0041	0.1329	1.3851	52.13^‡^
Gold	0.0003	0.0578	-0.0511	0.0113	-0.3643	4.2084	478^†^
VIX	0.0012	0.4802	-0.2662	0.0876	1.2519	4.2527	638^‡^

Notes: The table shows descriptive statistics for the log returns of the four assets. Max., Min., Std.Dev and J-B. are maximum, minimum, standard deviation, and Jarque–Bera test respectively.

^‡^ indicates a rejection of the null hypothesis at the 1% significance level.

The USDX can be used to measure the credibility of dollar assets to other countries and the value of U.S. sovereign risk. Therefore, the higher the correlation between the US stock index and USDX, the lower the dependence of other countries on the US dollar. This is confirmed by the expansion of the hedge ratio during the post-COVID-19 period. Thus, after COVID-19, the dollar is more effective in hedging against the S&P 500 than against stock indices from other countries. This could also indicate that the credibility of non-dollar currencies has increased relative to the dollar, meaning that the status of non-dollar currencies slightly increased after COVID-19. For example, in developing countries with fiscal problems, the price of that country’s stock index is correlated with and affected by the exchange rate of that country’s currency against the price of the dollar, not the price of the USDX.

Furthermore, because the USDX is a significant hedge against the S&P 500 and not the Nikkei225 or EuroStoxx 50, it provides a new interpretation for the influence of the dollar. Future research should explore the degree of interdependence and the impact of the USDX. Accordingly, we propose a study on how the USDX is a valuable asset to the S&P 500 as a hedging asset and from the perspective of other countries’ currencies, how the US dollar provides stability to U.S. market flows, and how they influence each other.

The findings of this study have several important implications. First, both the optimal portfolio weight of the USDX and its volatility are smaller than those of the other assets, as shown in Table 5. This indicates that the USDX is an attractive hedge against the S&P 500 index. Therefore, the USDX can be used as a low-risk hedge to construct an optimal portfolio, making it a highly appealing product for hedging the S&P 500 index.

Second, the hedge ratio for the USDX increased significantly during the COVID-19 period. This implies that a larger amount of hedge capital must be allocated to the USDX than to the VIX or gold. Furthermore, this increased hedge ratio highlights the greater interdependence and influence between the USDX and S&P 500 index, especially in the context of the COVID-19 pandemic. This observation is consistent with the findings presented in Tables 5 and 9, which show that despite the USDX being a lower-risk and lower-volatility asset compared to VIX and gold, its hedge ratio has increased following the onset of COVID-19. Thus, the correlation between the USDX and S&P 500 index has strengthened, reflecting their heightened interdependence.

Third, Table 9 provides descriptive statistics, indicating that the USDX exhibits very low volatility. Consequently, if a low-risk portfolio can be constructed by incorporating the USDX, it holds significant value as a hedge asset. This implies that the USDX can form part of a lower-risk portfolio even though it is less risky than other assets. This characteristic further enhances the USDX’s attractiveness as a hedging instrument.

To date, the safety of USDX as an asset class in the U.S. stock market has received limited academic attention. Our study demonstrated the USDX’s potential to serve as a safe haven asset within the S&P 500 Index, providing valuable investment insights for market participants, particularly those interested in the U.S. stock market.

As a future research topic, we propose an analysis of the hedging performance of the USDX during a period of turmoil in the US stock market caused by the RU war. This analysis would aim to evaluate how effectively the USDX serves as a hedge under such circumstances. Additionally, we suggest comparing the hedging performance of the USDX with other assets commonly used for hedging, such as cryptocurrencies, gold, silver, and the VIX. By conducting this study, we can enhance our understanding of the USDX’s hedging performance and its comparative advantages and disadvantages over other hedging assets. This knowledge can be valuable for investors and financial institutions seeking reliable hedging instruments during times of market uncertainty.

## US dollar index(USDX)

The US index (USDX) is defined as

$$\begin{array}{ccc} \text{USDX} & = & C\times {\text{EURUSD}}^{-0.576}\times {\text{USDJPY}}^{0.136}\times {\text{GBPUSD}}^{-0.119}\\ & & \times {\text{USDCAD}}^{0.091}\times {\text{USDSEK}}^{0.042}\times {\text{USDCHF}}^{0.036} \end{array}$$ (7)

where *C* = 50.14348112 and EURUSD, USDJPY, GBPUSD, USDCAD, USDSEK, and USDCHF are weighted. Such weight was established in 1973. EURUSD is a pair of the EU currencies: euro (€) and USD. USDJPY is a pair of Japan’s currencies: Japanese Yen(¥) and USD. GBPUSD is a pair of the United Kingdom’s currencies: pound (£) and USD. USDCAD is a pair of Canada’s currencies: Canadian dollar ($) and USD. USDSEK is a pair consisting of Sweden’s currency krona(SEK) and USD. USDCHF is pair of Swiss currency francs (CHF) and USD. USDX’s weight changed when the EU was founded. The original weights included West German marks, French franc, Italian lira, Dutch guilder, and Belgium franc. The euro combines the currencies of all five countries. There are no scheduled adjustments or rebalancing in USDX. The only adjustment and rebalance was executed at the introduction of the euro. Therefore, it has doubt about representing the dollar’s value across the world. Thus, the U.S. Federal Reserve created a trade-weighted US dollar index reflecting the volume of modern pairs traded with the dollar. However, the USDX has its own future trade contract; therefore, its mass popularity is high.

## VAR lag order selection criteria

Table 2.
